# The use of a severity index to analyse impact of bacterial zoonoses on welfare of wildlife populations

**DOI:** 10.1017/awf.2025.10024

**Published:** 2025-07-22

**Authors:** Kristen Hirst, Samniqueka Halsey

**Affiliations:** Department Natural Resources, University of Missouri, Columbia, MO, United States

**Keywords:** Animal welfare, bacteria, pathogen, severity index, wildlife, zoonosis

## Abstract

Increasing disease outbreaks and declining biodiversity underscore the need for understanding the impact pathogens have on wildlife populations. To understand how zoonoses impact wild animal welfare, we created a severity index. Using signs of disease information from a bacterial zoonotic disease database, we quantified severity of each sign of disease combined with the number of welfare domains and body systems the pathogen impacts to find the severity index value (SIV) of each unique host-pathogen relationship. We then investigated the effects of host-pathogen richness and conservation status against SIV. We found there to be a strong, negative correlation between increasing pathogen richness and SIV. Species of least concern (LC) were not significantly more likely to have higher SIV than species of conservation concern (CC), but CC species did not have a significant decline of SIV with increasing pathogen richness. This study provides an insight into the relationships between pathogen richness and the risk of pathogen infections to wildlife.

## Introduction

In the last few decades, outbreaks of major zoonotic pathogens, such as zika virus, Ebola virus, avian influenza and others, have emphasised the need for a better understanding of zoonotic pathogens (Bermejo *et al.* 2006; Wang *et al.* 2016; Calle-Hernández *et al.* 2023). Zoonoses are recognised as posing a serious threat to humans, domestic animals, and wildlife, and efforts to monitor and mitigate outbreaks are a priority (EFSA *et* *al.* 2023). Disease outbreaks can impact a variety of wildlife species and have devasting impacts on wildlife populations. For example, the H5N1 strain of avian influenza has undergone an expansion in geographic range, impacting bird populations throughout 30 countries and has even started to cause massive population declines in non-avian populations, such as the South American sea lion (*Otaria flavescens*) in Chile (Kandeil *et al.* 2023; Ulloa *et al.* 2023). It is well-known that zoonoses can cause population declines, but there remains a paucity of research regarding how pathogens impact the welfare of wild animals.

Animal welfare is defined in numerous ways however welfare is generally considered to comprise the state of the animal’s body and mind (Hewson *et al.* 2003). Pathogen infection can both negatively impact an animal’s health as well as cause changes in the behaviour of animals. In domestic animals, it has been observed that poor living environments or diets may be associated with stress, clinical disease, and economic losses. (Humphrey 2006). When attempting to conserve wild animals, animal welfare should be an important consideration when implementing management practices. Methods of conservation, such as translocation and reintroduction programmes, can increase stress levels in animals which may reduce immune system function and increase disease susceptibility, therefore lowering the chances of successful intervention if welfare is not considered (Teixeira *et al.* 2007). Practices that fail to account for animal welfare may result in unsuccessful management attempts and cause further harm to wild populations.

Unfortunately, investigating animal welfare in wildlife can be difficult and rarely attempted. Traditional animal welfare practices observe the individual’s needs. Conservationists tend to observe the needs of wildlife in terms of populations and species, making it difficult to gain a true understanding of wild animal welfare (Beausoleil 2014). Historically, animal welfare has not been a major component of wildlife management practices, and the welfare standards applied to domestic and commercial animals are not required by wildlife managers (Hampton & Hyndman 2019). Despite these difficulties, more efforts are being made to incorporate animal welfare into wildlife management and monitoring (Clegg & Delfour 2018; Harvey *et al.* 2020). One method for evaluating welfare in animals, domestic and wild, is to use the Five Domains of animal welfare. These were first proposed in 1994 and been adapted over the years to incorporate shifting viewpoints and ethical ideals of animal welfare (Mellor & Reid 1994, Mellor *et al*. 2020). The Five Domains comprise five categories which are evaluated to obtain an overall impression of an individual’s welfare: Nutrition, Health, Behaviour, Environment, and Mental state (Table 1; Mellor & Beausoleil 2015). Welfare is negatively impacted by disease, but the extent to which it affects welfare depends on a host of factors. Disease can result in compromised welfare in individuals and may lead to premature death (Teng *et al.* 2018).

**Table 1.** The Five Domains of animal welfare. A general description of each domain, as well as examples that could impact each domain. Based on previously published works (Mellor *et al.* 1994; Mellor & Beausoleil 2015; Mellor *et al.* 2020)

Domains	Description	Examples
Physical domain		
Nutrition	Dependent on access to appropriate amount of food and water	Water intake, Food intake, Food/water quality
Environment	Dependent on tolerability to environmental and artificial atmospheric and physical challenges	Thermal extremes, Pollutants, Unpleasant odours, Light intensity, Noise level, Unpredictable events
Health	Dependent on disease or injury	Fitness level, Illness, Functional impairment, Poisoning
Behaviour	Dependent on behaviour, movement restrictions, predator-prey interactions	Reproductive capacity, Animal-animal interactions, Threat avoidance, Stimulating environment, Movement
Affective experience domain		
Mental state	Dependent on the other four domains, the overall comfort of the individual	Thirst, Hunger, Anxiety, Fear, Pain, Boredom, Loneliness

Applying animal welfare to disease is still a relatively new idea. A recent study used expert advice to assess severity of signs of disease in combination with duration and frequency of illness to evaluate pathogen severity in domestic pigs and cattle (Nielsen *et al.* 2021). Assessing welfare in diseased wildlife is even more challenging. Often, we only have a snapshot of how disease impacts the individual and are unable to follow how the disease progresses, unlike with domestic animals. A recent study sought to fill in some of these gaps with an investigation of *Mycobacterium ulcerans* infection in common ringtail possums (*Pseudocheirus peregrinu*s). Welfare was incorporated as a metric for determining the severity of the pathogen for this species, alongside clinical and necropsy findings (Hobbs *et al.* 2024). Identifying ways to measure the severity of disease on wildlife will provide an insight into appropriate management strategies for different wildlife diseases and wildlife populations.

We set out to investigate how welfare is impacted by pathogen infection by creating a severity index that measures how pathogens impact the host physically, and how the pathogens impact the welfare of the host species, using the Five Domains as a framework for animal welfare assessment. We used previously published data reporting clinical signs of disease in wildlife and captive individuals and attempted to measure the impact of bacterial zoonoses on their mammalian, avian, and reptilian wildlife hosts. We then applied this welfare assessment method to explore groups of animals that are more or less likely to be impacted by bacterial zoonoses. An assessment of host-pathogen richness, defined as the number of known pathogens a host can carry, found certain taxonomic orders show a higher pathogen richness than others (Shaw *et al.* 2020). The aim was to use severity scores to investigate the relationship between host-pathogen richness and pathogen severity. In addition, we explored how pathogen severity differs between species of least concern (LC) and those of conservation concern (CC). There is increasing evidence that disease plays a role in the decline of threatened and endangered species and inhibits population recovery (Brand 2013). Understanding how pathogens impact the host will provide an insight into how wild populations are impacted by disease outbreaks and may help us predict the impact future outbreaks will have on wild populations.

## Materials and methods

We used the Bacterial Zoonosis and Clinical Symptoms in Wildlife database to evaluate the severity of individual host-pathogen relationships (Hirst & Halsey 2023). The database contains detailed information on bacterial diseases in mammal, bird, and reptile species of wildlife. To evaluate disease severity, we analysed three variables: mean symptom severity (*S*), body system count (*B*), and animal welfare count (*D*; Eq.1; Table 2).

**Table 2.** Description and abbreviation of variables used to calculate severity index value (SIV) scores

Symbol	Description	Range
*S*	Mean of summed severity score of pathogen signs of disease	1–3
*B*	Count of host body systems impacted by pathogen	1–11
*D*	Count of host welfare domains impacted by pathogen	1–4
*SIV*	Severity index value, calculated using the following equation: *SIV = (S × B)^D^*	> 0

Assessing severity of disease in animals is difficult since animals are unable to tell us how much pain or discomfort they are in. We approached this challenge by scaling the severity for each symptom, then taking the mean of the total scores for each pathogen relationship. Our methods were a modification of the cumulative illness rating scale (CIRS) as proposed by Linn *et al.* (1968). The CIRS framework seeks to assess severity of disease based on ranking the signs of disease on a 0–4 scale (Hudon *et al.* 2007). This method does not require patient input to assess severity, which makes applying it to wildlife optimal. Our severity rank assessment contains only three ranks which were assigned based on the extent to which the sign of disease is likely to impact normal host behaviours. The greater the impact on normal behaviours, the greater the capacity of the animal to suffer. The descriptions of the ranks of clinical signs are as follows:

1. Sign has no or mild morbidity regarding individual’s health/welfare (i.e. coughing, mild lesions, nasal discharge, etc). Individual’s normal behaviours tend not to be impaired and symptom highly unlikely to lead to mortality.
2. Sign causes moderate morbidity on individuals’ health/welfare (i.e. moderate lesions, inappetence, depression, etc). Individuals may not be able to behave as normal and are placed at a greater risk of mortality.
3. Sign causes severe morbidity on individuals’ health/welfare. Individual unable to demonstrate normal behaviour and mortality will most likely occur (i.e. severe lesions, paralysis, organ failure, etc).

Once each sign of disease was ranked, the scores were totalled, and the mean symptom severity was calculated.

The body system count was calculated as the sum of the body system categories the pathogen impacts upon infection. The eleven body systems are: integumentary, muscular, skeletal, cardiovascular, respiratory, gastrointestinal, urinary, endocrine, nervous, lymphatic and reproductive. This number ranged from 1–11.

The animal welfare count was derived by totalling the number of the Five Domain welfare categories the pathogen impacts upon infection. The Five Domains comprises of the following categories: Nutrition, Health, Environment, Behaviour, and Mental state. For a welfare domain to be included, a pathogen would have to produce a sign of disease that directly or indirectly impacted that domain. For example, pathogens that impact nutrition could have hosts that exhibited symptoms such as emaciation, vomiting, dehydration, etc. The environmental domain refers to the area around the animal’s natural habitat. The condition of the host’s environment can increase frequency and intensity of diseases and is a relevant factor when assessing disease risk. However, we are primarily interested in the effects of the pathogen on the host’s welfare, and since the pathogen cannot directly alter the environment around the host, we excluded it from welfare counts. Consequently, the animal welfare count ranged from 1–4.

Using these variables, we created the following equation to calculate the severity index value (SIV):

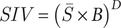



**Figure 1.**
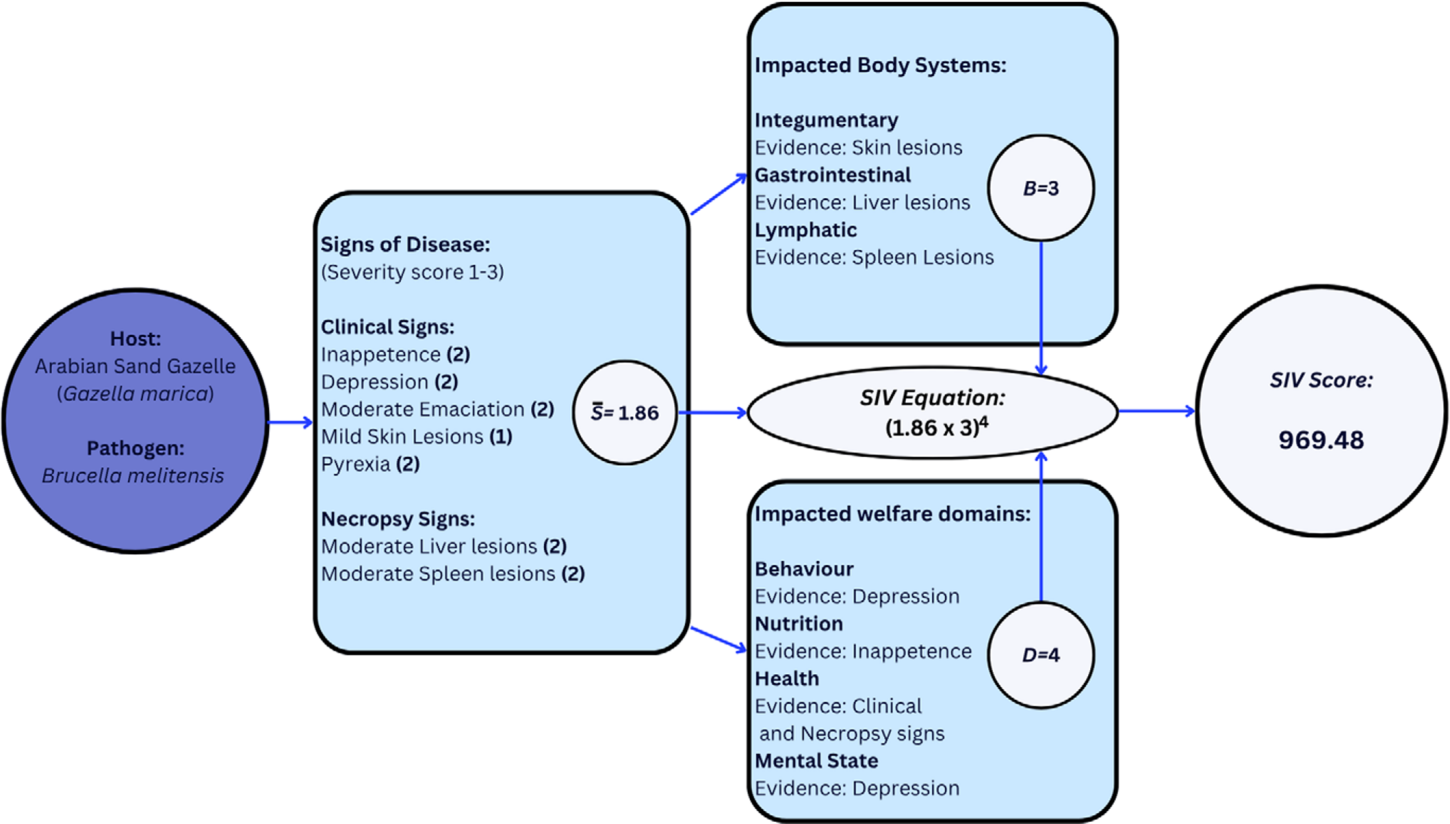
Example of severity index scoring in the Arabian sand gazelle (*Gazella marica*). Each symptom is scored on a scale of 1–3 and the mean score is taken for overall severity score. Lesions were observed in the skin, liver and spleen covering three different body systems. Depression impacts animal behaviour, inappetence and emaciation impacts an animal’s nutrition, clinical signs causing an animal to feel sick impact an animal’s mental state and overall health making the welfare count 4. The overall SIV value for this animal with this pathogen is 969.48 (Soares *et al.* 2019).

This formula integrates key aspects of disease severity by combining symptom (*S*), systemic involvement (*B*), and welfare impact (*D*) in a biologically meaningful way. Multiplying *S* and *B* accounts for both the severity of clinical signs and the breadth of physiological involvement, while raising the product to the power of *D* reflects the compounding effect of multi-domain welfare impacts. This structure ensures that diseases affecting multiple systems and welfare domains are weighted more heavily, aligning with observations that broader physiological and welfare disruptions exacerbate overall disease burden non-linearly. This calculation was applied to each host-pathogen relationship in the database for which signs of disease were available. An example of the process for identifying severity index value is shown in Figure 1.

### Data analysis

We aimed to determine how host/species bacterial richness and conservation status impacted pathogen severity. Conservation status is assigned based on the assessed current and future health of populations. Conservation status was assigned to species according to the International Union for Conservation of Nature’s Red List of Threatened Species (IUCN 2022). To determine the impact of zoonotic bacterial richness on disease severity in wildlife, we assessed how pathogen richness (count) impacted the severity index. We then investigated the relationship between conservation status and severity index value, excluding species without a conservation status. Although the data failed to meet the test of normality, due to the large sample size of our dataset and the ANOVA being robust to departures, we proceeded with the ANOVA analysis (Blanca *et al.* 2017). For the best-supported model, we then used a simple main effect test to determine the significance of pathogen richness and conservation status against SIV scores. Adjusted *P*-values (< 0.05) with the Bonferroni method *post hoc* were used to identify the significant comparisons. All analyses were run using R 2022.07.1 (R Code Team 2022).

## Results

Signs of pathogen infection were recorded in 2,588 of the 4,971 host-pathogen relationships registered in the Bacterial Zoonosis and Clinical Symptoms in Wildlife database (Hirst & Halsey 2023). In total, pathogen SIVs ranged from a minimum score of 1 for asymptomatic carriers (n = 1,117) to a maximum score of 324,645.7 belonging to a harbour porpoise (*Phocoena phocoena*) infected with the pathogen *Staphylococcus aureus.* The mean (± SD) overall score for pathogen severity is 2259.854 (± 10,191.92). Sixteen outliers were excluded since the severity values exceeded 50,000.

**Figure 2.**
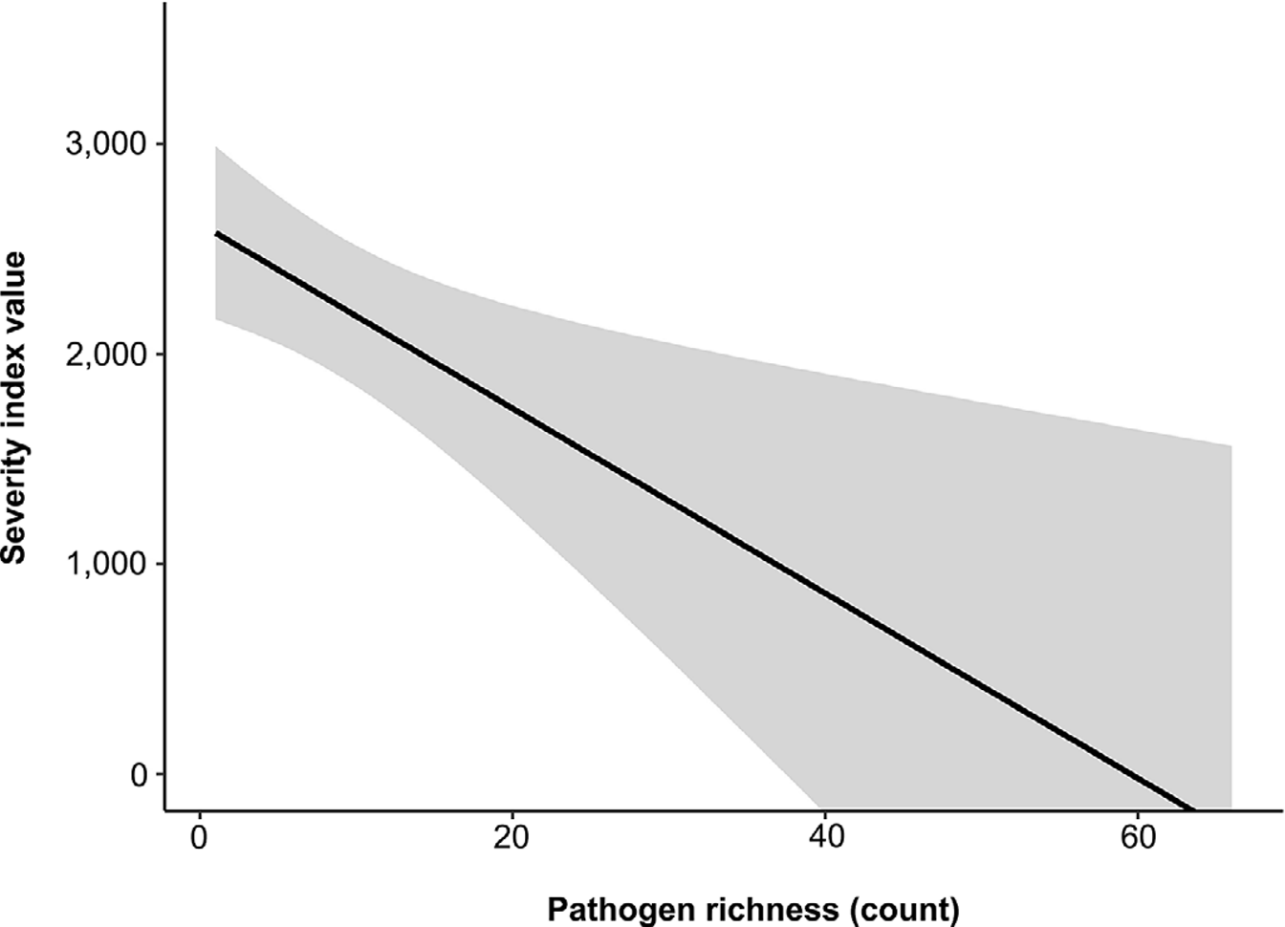
Displays a linear regression comparing host severity index value (SIV) and zoonotic bacterial richness.

**Figure 3.**
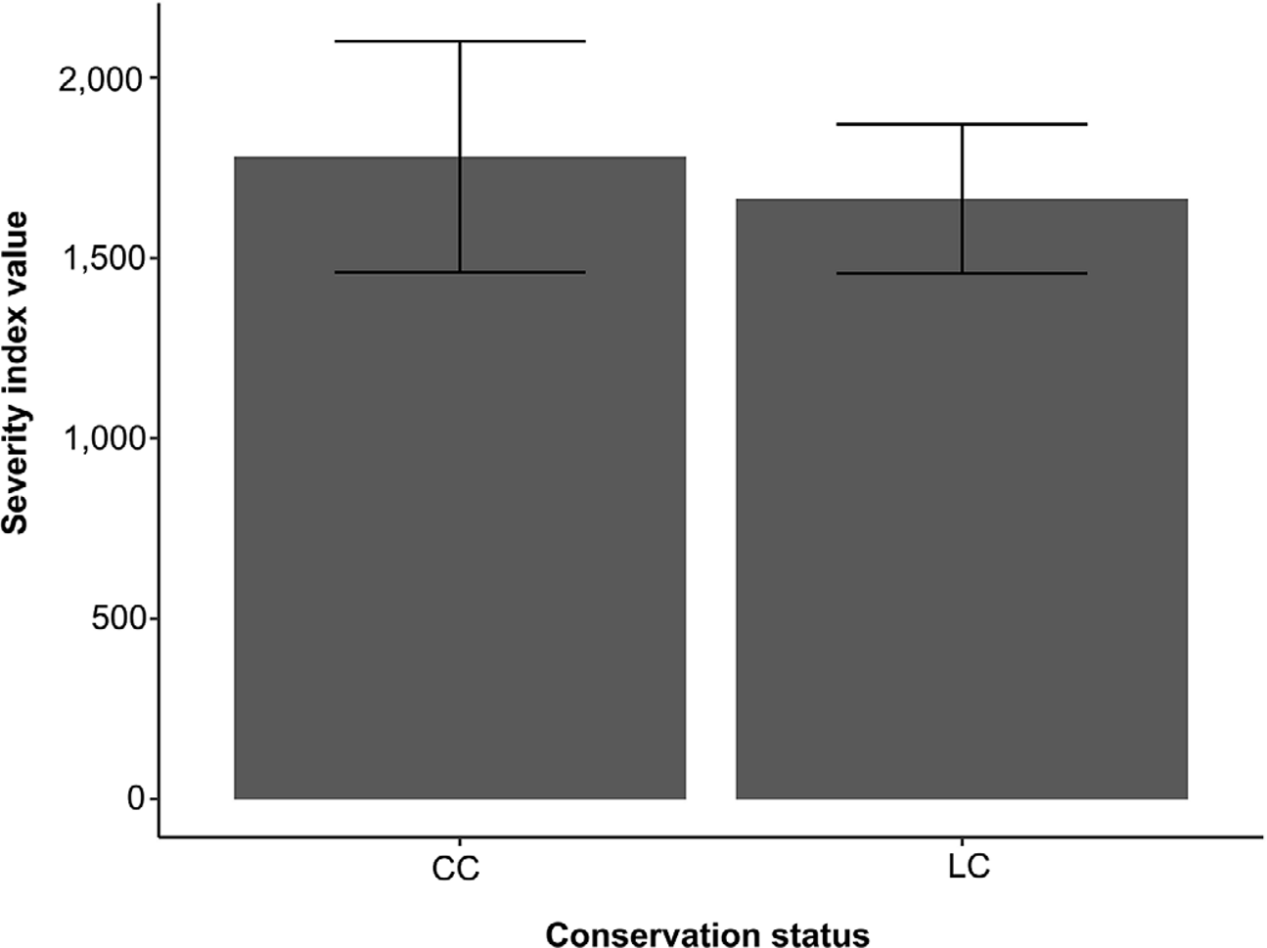
An analysis of variance comparing host species IUCN status of conservation concern (CC) and least concern (LC) and severity index value (SIV).

We found bacterial pathogen richness to show a negative correlation with pathogen SIV (r^2^ = 0.01989; *P* < 0.001). Greater SIV scores were commonly observed in species that hosted fewer zoonotic bacterial pathogens (Figure 2). When comparing SIV scores between conservation statuses, we found species of conservation concern (CC) to have a greater average SIV score, but not to the extent that differed significantly from species of least concern (LC) (F_1,2499_ = 0.223; *P* = 0.637; Figure 3).

**Figure 4.**
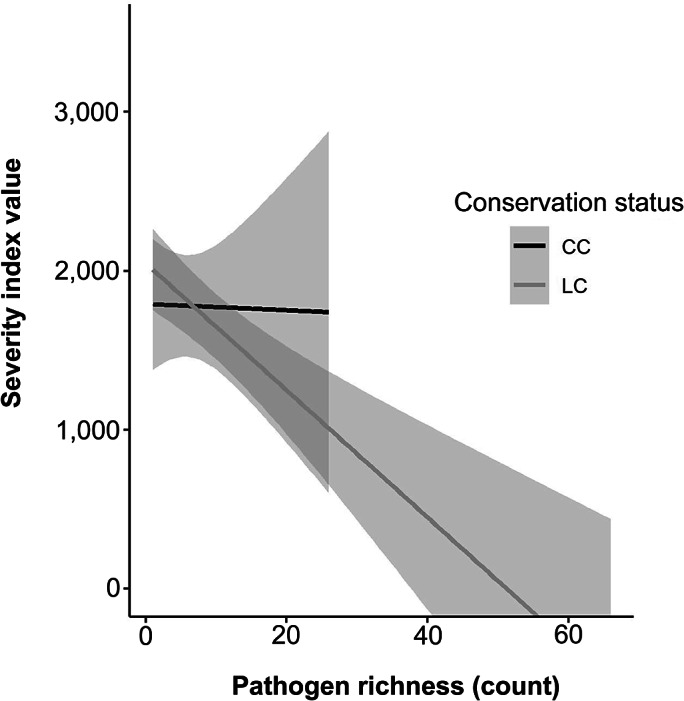
An analysis of co-variance comparing severity index value to host bacterial species richness and with an IUCN status of conservation concern (CC) and least concern (LC).

Increased pathogen richness was found to result in a decreased SIV score for wildlife species (F_1,2498_ = 13.265; *P* < 0.001); with no difference between conservation status and SIV score (F_1,2498_ = 0.0104; *P* = 0.9189; Figure 4). A main effect *post hoc* test showed that pathogen richness in LC species correlated significantly with SIV score (F_1,1889_ = 13.5; *P* < 0.001) but pathogen richness in CC species was not significantly correlated with SIV score (F_1,608_ = 0.004; *P* = 0.952).

## Discussion

With pathogen outbreaks increasing in frequency and both distribution and biodiversity continuing to decline, the need to understand the impact of pathogens on wildlife populations is more important than ever (Daszak *et al.* 2000; Chala & Hamde 2021). Zoonoses account for more than 60% of infectious diseases and as much as 75% of emerging infectious diseases and can have major impacts on wildlife populations (Asokan & Asokan 2016). Animal welfare is rarely observed in wildlife populations, and disease impacts on welfare can be challenging to observe.

Pathogen infections are not always apparent; in most cases, the disease is not considered a factor in species declines until carcases are found (Ethier *et al.* 2016). When disease outbreaks are apparent, practical methods for evaluating welfare involve quantifying the number of individuals involved and the duration of the disease event but assessing the severity of harm and capacity for animals to suffer is a more abstract concept (Kirkwood *et al.* 1994). There are protocols for evaluating animal welfare in wildlife populations (Harvey *et al.* 2020), but large-scale analysis of disease severity is lacking. Using the Bacterial Zoonosis and Clinical Symptoms in Wildlife database we quantified the severity of disease signs for each host-pathogen relationship and incorporated animal welfare impacts to measure the severity of disease. We used these SIV scores to analyse the relationship between host-pathogen richness, conservation, and disease severity.

We found a significant correlation between bacterial host-pathogen richness and pathogen SIV whereby pathogen SIV decreased in species showing greater bacterial pathogen richness. The greater pathogen richness in individual species not displaying negative impacts of disease could make them an ideal candidate for reservoir species monitoring. Rodents are common host species that can hold two or more pathogens and have been investigated for their potential as reservoir species (Han *et al.* 2015). Individuals identified here with high pathogen richness and low disease severity scores could be optimal candidates for emerging infectious disease surveillance in wildlife.

Surveying species with high pathogen richness could allow for the pre-emptive discovery of emerging zoonotic bacteria. Sentinel surveillance has recently been proposed and used to monitor a variety of zoonotic pathogens (Komar 2001; Aguirre 2009; Wood *et al.* 2012). Monitoring of individual species may not be geographically, economically, or physically feasible, particularly when trying to detect new diseases, but monitoring larger taxonomic groups would provide more options for investigation and could provide a broader understanding of pathogen presence. In addition, consistent monitoring efforts could fill in some of the gaps of disease impacts on wildlife, such as symptoms of infection, disease progression, and severity of illness. This information would then be able to be used to supplement our understanding of welfare impacts in wildlife populations.

We found that SIV scores of species of least concern (LC) did not differ significantly from those of conservation concern (CC), but a *post hoc* analysis of LC and CC combined with pathogen richness found LC species to show a significant negative linear regression with increasing pathogen richness, while CC species did not. Species of conservation concern can be at greater risk from pathogen infection due to decreased genetic diversity in immune systems as a result of smaller population sizes (O’Brien & Evermann 1988). Decreased welfare in wildlife can lead to increased stress levels, which can have negative impacts on the fitness and reproduction of wild animals (Edwards *et al.* 2019). Our results indicate that the severity of disease is not greater in CC species compared to LC species, however, pathogen severity did not decrease with CC species as it did with LC species.

This could mean CC species are at greater risk of severe disease compared to LC species even with increasing pathogen richness, potentially due to factors such as a lack of genetic diversity and compromised welfare. However, we cannot discount biases in the data as a reason. Overall, there were 3,806 LC host-pathogen relationships and 1,084 CC host-pathogen relationships. Of the CC species in which host-pathogen relationships were clinically described, 47% were from captive situations. In contrast, only 15% of LC species were from captive situations. Captive populations have more contact with humans and are typically exposed to more stress than their wild counterparts, thereby increasing their susceptibility to diseases, exacerbating clinical signs of diseases, and potentially introducing them to pathogens they would not otherwise have experienced in free-range situations. They are also more heavily monitored, illnesses are tracked and treated, with the overall impact of the disease recorded in clinical reports. Since more signs of disease and welfare information are available in captive situations, it may have exacerbated some of the SIV scores. Evaluating disease severity in wildlife is limited and generally applied to species undergoing attempts at reintroduction or populations of conservation concern (Corn & Nettles 2001; Kilbourn *et al.* 2003; Gaydos *et al.* 2004).

There were also differences in pathogen richness between LC and CC species. The maximum pathogen richness in CC populations belonged to the lion (*Panthera leo*) which had 26 different bacterial pathogens, while in LC species the species with the greatest pathogen richness was the wild boar (*Sus scrofa*; n = 66). There was variation between pathogen richness, and it is unknown whether species with more pathogens have less severity or if this result was due to a few outliers or a lack of information on host-pathogen relationships. Our analysis offers an insight into the relationship between pathogen severity and pathogen richness, but in order to fully understand the impact, more information is needed regarding host response to pathogen infections in all wildlife and pathogen relationships, not merely those relating to bacterial zoonoses.

Overall, only 52% (n = 2,588) of the host zoonotic bacterial disease database contained information on pathogen signs in host species. Many of these reports were from single cases of captive individuals and would likely impact wildlife populations differently. Signs of disease are often incidental findings in wildlife investigations, so the complete disease progression is likely lacking for many of these cases. It should be emphasised that this severity index is based only on factors concerning animal welfare status. Therefore, severity in this case reflects the overall health of the host during infection and is not dependent on death rates caused by infection. In fact, diseases that cause acute or peri-acute death in host species will have lower SIV values than those that are more chronic because signs of disease and welfare status can be observed with animal morbidity more often than mortality. Welfare status cannot be obtained when an animal is found post mortem; in these cases, SIV scores were analysed based primarily on necropsy findings and can likely misrepresent the true severity of some host-pathogen relationships.

Time, or duration of the pathogen infection, and likelihood of infection are additional characteristics we are lacking. Duration of infection is important when analysing impacts to welfare (Nielsen *et al.* 2021). As mentioned above, most case reports listed here occur post mortem or are only observed during the acute phase of the disease since that is when the infection is most likely to be observed. Captive species were more likely to observe the full progression of the disease, but typically treatment was attempted which skews the natural duration of the illness. As a result, only a small handful of well-documented cases had accurate descriptions of the duration of pathogen infection. We opted, therefore, to exclude time as a factor. Likelihood of infection was not incorporated into the severity index as there was a lack of information for many of the pathogens on this metric. Likelihood of infection for the more well known or clinically important diseases could be assessed, but as this database includes several more obscure pathogens an accurate assessment of likelihood of infection was not feasible and therefore not attempted. Future research would benefit from incorporating time and likelihood of infection as factors and would lead to further insights into how these pathogens impact the welfare of wildlife populations.

### Animal welfare implications

Wildlife disease severity is often associated with the number of deaths that occur in populations due to infection. However, the number of deaths a disease causes is not the only factor that influences population health and welfare. By incorporating animal welfare as a measure of disease severity in the severity index, we can assess the broad impact disease has on wildlife populations and identify areas of wildlife disease research and management to target in the future.

## Conclusion

We assessed the impact of bacterial zoonotic pathogens on wildlife hosts with specific emphasis on animal welfare. To classify as a zoonosis, a pathogen must impact at a minimum two host species, a human and an animal, and the severity of the disease can vary depending on the host species. We found a strong correlation between increasing host pathogen richness and decreasing severity of disease across wildlife species. We believe that a host’s ability to carry many pathogens with little impact to itself makes a host species a viable candidate for wildlife disease monitoring, and further investigation of these species as competent reservoir hosts is warranted. Species of conservation concern did not exhibit a significant relationship with SIV score, but species of least concern did. This finding is concerning due to the at-risk nature of CC populations and warrants further investigation. In addition, although we found no difference in disease severity between species of conservation concern and those of least concern, species of conservation concern, on average, have higher severity values. We suspect that this might be due to either a low sample size or research biases and also merits further investigation. With biodiversity continuing to decline we need a better understanding of factors that can negatively impact wild animal welfare as it is a contributing factor to the overall health and stability of wildlife populations.

## Data Availability

Original datasets are available in a publicly accessible repository: Dryad Digital Repository, https://doi.org/10.5061/dryad.n5tb2rc12.
